# IRF4 as an Oncogenic Master Transcription Factor

**DOI:** 10.3390/cancers14174314

**Published:** 2022-09-02

**Authors:** Regina Wan Ju Wong, Jolynn Zu Lin Ong, Madelaine Skolastika Theardy, Takaomi Sanda

**Affiliations:** 1 Cancer Science Institute of Singapore, National University of Singapore, Singapore 117599, Singapore; 2 Department of Medicine, Yong Loo Lin School of Medicine, National University of Singapore, Singapore 117599, Singapore

**Keywords:** IRF4, oncogene, transcription factor, mature lymphoid neoplasms

## Abstract

**Simple Summary:**

Master transcription factors regulate essential developmental processes and cellular maintenance that characterize cell identity. Many of them also serve as oncogenes when aberrantly expressed or activated. IRF4 is one of prime examples of oncogenic master transcription factors that has been implicated in various mature lymphoid neoplasms. IRF4 forms unique regulatory circuits and induces oncogenic transcription programs through the interactions with upstream pathways and binding partners.

**Abstract:**

IRF4 is a transcription factor in the interferon regulatory factor (IRF) family. Since the discovery of this gene, various research fields including immunology and oncology have highlighted the unique characteristics and the importance of IRF4 in several biological processes that distinguish it from other IRF family members. In normal lymphocyte development and immunity, IRF4 mediates critical immune responses via interactions with upstream signaling pathways, such as the T-cell receptor and B-cell receptor pathways, as well as their binding partners, which are uniquely expressed in each cell type. On the other hand, IRF4 acts as an oncogene in various mature lymphoid neoplasms when abnormally expressed. IRF4 induces several oncogenes, such as *MYC*, as well as genes that characterize each cell type by utilizing its ability as a master regulator of immunity. IRF4 and its upstream factor NF-κB form a transcriptional regulatory circuit, including feedback and feedforward loops, to maintain the oncogenic transcriptional program in malignant lymphoid cells. In this review article, we provide an overview of the molecular functions of IRF4 in mature lymphoid neoplasms and highlight its upstream and downstream pathways, as well as the regulatory circuits mediated by IRF4.

## 1. Introduction

Interferon regulatory factor (IRF) family proteins were originally identified as a group of DNA-binding proteins that can be induced upon type I interferon (IFN) signaling [1,2]. Other stimuli, such as type II IFNs, cytokines, and viral infection, were later found to induce and activate these proteins [3,4]. Notably, IRF4, a member of the IRF family, possesses several distinct characteristics that distinguish it from other IRF family members. For example, *IRF4* is not induced by type I or II IFNs; instead, it is induced upon activation of T-cell receptor (TCR) signaling in T cells [4], B-cell receptor (BCR), IL-4, and CD40 signaling in B cells [5,6], and Toll-like receptor (TLR) signaling by lipopolysaccharide (LPS) in macrophages. Additionally, unlike other IRF family members, the expression of *IRF4* is largely restricted to immune cells, namely, lymphoid cells, monocytes, macrophages, and dendritic cells (DCs) [4,5,7,8].

It is also noteworthy that different names were given to this gene in early studies, highlighting the importance of IRF4 in different biological fields. For example, IRF4 was described as “*lymphocyte-specific interferon regulatory factor*” (*LSIRF*) on the basis of its expression pattern [4]. IRF4 was also named “*multiple myeloma oncogene 1* (*MUM1*)” [9] and “*interferon consensus sequence-binding protein in adult T-cell leukemia cell lines or activated T cells* (*ICSAT*)” [10] in the context of cancers. Additionally, IRF4 was originally identified by molecular biological approaches as a PU.1-interacting protein that binds to the κE3’ enhancer element and EM5 DNA motif, thus being named “*PU.1-interacting partner*” (*Pip1*) [11] and “*nuclear factor which binds to EM5 element*” (*NF-EM5*) [12]. Thus, different biological fields have highlighted the unique characteristics and importance of IRF4.

## 2. IRF4 as a Master Regulator of Immunity

IRF4 has been implicated as a master regulator of immunity, critically mediating the differentiation, affinity maturation, and function of T cells, B cells, macrophages, and DCs (please also refer to a previous review article by us for more details) [13]. The phenotype of *Irf4* knockout mice was first described in 1997; these mice exhibited attenuated cytotoxicity of T cells and immunoglobulin (Ig) production [8]. A number of studies subsequently demonstrated the specific functions of IRF4 in each immune cell type.

Notably, IRF4 is expressed in mature single-positive (SP) T cells and plays a pivotal role in T-cell fate dynamics through its interaction with various transcriptional cofactors in a cellular context-specific manner. The TCR pathway is the major upstream signaling pathway that regulates IRF4 in T cells, and TCR stimulation induces IRF4 expression in a signal strength-dependent manner [14,15,16,17]. In general, CD4^+^ SP T-cell differentiation is driven by antigen-specific stimulation and different types of cytokines. In this regard, IRF4 mediates the response to IL-4 and determines Th1 and Th2 development by interacting with other transcription factors, i.e., T-BET and GATA3, respectively [18,19]. Meanwhile, IRF4 interacts with ROR-γt to direct the differentiation of naïve T cells to Th17 cells and BCL-6 for the differentiation to T follicular helper cells. IRF4 also cooperates with BLIMP1 or FOXP3 to induce the differentiation of effector regulatory T cells (Tregs), suppressing Th1 and Th2 immunity [20]. The cytotoxic activity of CD8^+^ SP T cells is also highly governed by IRF4. *Irf4*-deficient mice show undetectable levels of antigen-specific CD8^+^ T cells, and, in these mice, immune challenge with influenza virus or lymphocytic choriomeningitis virus results in abolished effector cell marker expression and responses [8,14]. A mechanistic study showed that IRF4 is a central regulator of metabolic activity in CD8^+^ T cells, particularly by sustaining aerobic glycolysis required to maintain clonal expansion and effector differentiation [14].

In addition to its roles in T cells, IRF4 plays major roles in the development and function of B cells in various stages. In general, in response to antigen challenge, B-cell development takes place in a stepwise manner consisting of affinity maturation, isotype switching, and terminal differentiation into antibody-secreting plasma cells. Notably, *Irf4*-deficient mice showed blockade at the late stage of peripheral B-cell maturation, together with the absence of germinal centers in B-cell follicles residing in the spleen and lymph nodes [8]. Consequently, these mice displayed no detectable levels of all Ig subclasses and hapten-specific antibodies. Combined loss of *Irf4* and *Irf8* results in the failure of pre-B cells to downregulate ectopic expression of pre-B-cell receptor (pre-BCR) complexes, which is required to terminate the cell cycle and induce *Ig* light-chain gene expression and rearrangement, thereby hindering the pre-B-to-B transition [21]. Additionally, the impairment of isotype switching arises in *Irf4* knockout B cells as a result of failure to induce *Aicda*, a gene encoding activation-induced deaminase (AID), which is critical in mediating somatic hypermutation and *Ig* class switch recombination [22,23,24]. IRF4 also promotes the terminal differentiation of B cells into plasma cells via the direct activation of the *PRDM1/Blimp-1* gene [24,25]. Additionally, *IRF4* deficiency leads to the migration of follicular B cells to the splenic marginal zone [26], revealing IRF4’s role in controlling B-cell positioning and the enrichment of genes responsible for migration and trafficking of marginal zone B cells. Retention of *IRF4*-deficient B cells is also associated with elevated expression and activation of NOTCH2, which is known to be indispensable for marginal zone B-cell lineage development [27,28].

IRF4’s roles have also been reported in other immune cell types, such as macrophages and DCs. IRF4 skews macrophages into the M2 phenotype by inducing IL-4 via the *JMJD3–IRF4* axis, competing with IRF5 for MyD88 binding to antagonize Toll-like receptor (TLR) signaling, suppressing M1 polarization, and promoting the secretion of IL-4 and IL-10 [29,30]. In DCs, IRF4 is vital in the development of specific DC subsets [31]. In particular, mice lacking *Irf4* exhibit normal proportions of conventional DC subsets in the thymus but not in the spleen, where there was a specific reduction in the number of splenic CD11b^high^CD4^+^CD8α cells [31]. This subset of DCs is responsible for inducing Th2-type responses. Another study also revealed that IRF4 directly activates the production of the Th2-stimulating cytokines IL-10 and IL-33 to promote DC^Th12^ differentiation and inflammation [32]. Taken together, these findings suggest that IRF4 plays major roles in immune cell development and function.

## 3. Molecular Functions of IRF4

### 3.1. IRF4 and DNA-Binding Motifs

IRF4 belongs to the IRF gene family, which encodes basic leucine zipper (bZIP)-type transcription factor proteins. bZIP proteins possess three modular functional regions: dimerization, DNA binding, and transcriptional regulation regions. More specifically, IRF family proteins contain a DNA-binding domain at their amino terminus, which recognizes multiple DNA-binding motifs in regulatory elements of certain genes, including interferon-stimulated response elements (ISREs) (5′–GAAAnnGAAA–3′ motifs) [4], Ets-IRF composite elements (EICE) (5’–GGAAnnGAAA–3’) [33], and AP-1-IRF composite elements (AICE) [34]. However, IRF4 needs to homodimerize or heterodimerize with different transcription factors depending on the type of immune cells for efficient binding and transcriptional regulation. Hence, IRF4 may play different roles depending on the cellular context, expressing different binding partners.

### 3.2. Binding Partners and Downstream Targets of IRF4

#### 3.2.1. ETS Family Transcription Factors

PU.1/SPI1, a hematopoietic ETS family transcription factor, is a notable binding partner of IRF4. In fact, IRF4 was first identified as a binding partner of this protein at the immunoglobulin κ3′ enhancer in B cells [12]. Binding with PU.1 was shown to release the carboxy-terminal autoinhibitory domain of IRF4 and increase its binding affinity, allowing the IRF4/PU.1 dimer to bind to the EICE motif [12,33]. Efficient interaction between IRF4 and PU.1 is facilitated by phosphorylation of the PEST domain of PU.1 at Ser148 [35]. Residues Arg232 and Arg235 in PU.1 are crucial for DNA recognition, while residues Arg88 and Cys99 in IRF4 are required for DNA binding [36]. The crystal structure of the PU.1/IRF-4/DNA ternary complex revealed that PU.1 and the IRF4 DNA-binding domains bound on opposite sides of the DNA and contorted the DNA into an “S shape” [36].

The IRF4–PU.1 complex regulates genes expressed in B cells, such as the Ig light chain [11,12] and CD20 [37], as well as macrophage-related genes during macrophage differentiation, including cathepsin C (CTSC), cystatin C (CST3), CSF-1 receptor/M-CSF receptor (CSF1R), scavenger receptor (MSR1) and Blimp-1 (PRDM1) [38]. IRF4 has also been found to heterodimerize with SPIB, an ETS family transcription factor that is closely related to PU.1, at EICEs in a manner similar to the IRF4–PU.1 interaction in B cells and myeloid cells [39].

#### 3.2.2. AP1 Family Transcription Factors

Although PU.1 and SPIB are not highly expressed in T cells or DCs, unlike in B cells, IRF4 also plays major roles in these immune cells, which express AP-1 family transcription factors such as BATF and JUN [40,41]. The cooperative binding of IRF4 with these proteins at AICEs has been reported. For example, a study reported that BATF binding is diminished in *Irf4*-deficient T cells, while IRF4 binding is diminished in the absence of *Batf* in T cells, confirming the cooperativity of these two transcription factors [40]. Similarly, another group demonstrated the inability of IRF4 to bind to DNA in the absence of BATF in DCs [41].

The interaction of these two proteins has been found to be involved in the pivotal role of IRF4 as a translator of TCR signaling [15]. In Th2 T cells, IRF4 conveys the magnitude of the TCR signal via the binding of the BATF–IRF4 complex to enhancers containing AICEs. The divergent TCR signaling outcomes depend on the affinity of those enhancers for the BATF–IRF4 complex. IRF4 can be induced in a dose-dependent manner to increase TCR signal strength [15]. Similarly, the level of BATF expression increases gradually depending on the magnitude of TCR signaling [15]. This event translates to parallel expression of known IRF4 target genes such as *GATA3* and *CTLA4* [15].

#### 3.2.3. Other IRF4-Interacting Proteins

Several other transcription factors, such as nuclear factor of activated T cells (NFATs), Foxp3, STAT3, STAT6, and IKZF1, have also been reported to interact with IRF4. In T cells, IRF4 can bind with NFATs to regulate inducible IL-4 gene expression [42]. The IRF4–Foxp3 complex has been reported to be crucial for the control of Th2 cell responses [43]. IRF4 has also been shown to bind and cooperate with STAT3 to facilitate IL-21-induced gene expression in T cells [44]. In addition, STAT6 can physically bind to IRF4 and drive the expression of IL-4-inducible genes in B cells [5]. More recently, IKZF1/IKAROS was identified as an IRF4-interacting partner that binds through zinc finger–IRF composite elements (ZICEs) during plasma cell differentiation [45].

### 3.3. Regulation of IRF4 Activity

#### 3.3.1. Upstream Signaling Pathways of IRF4

In the normal immune response, the expression and activity of IRF4 are strictly regulated at the transcriptional and post-transcriptional levels. Transcriptional regulation occurs in a stimulus (ligand)-dependent manner mediated by upstream signaling pathways, whereas, post-transcriptionally, it is achieved by phosphorylation and protein–protein interactions. Notably, these mechanisms vary among different immune cells. For example, in T cells, the *IRF4* gene promoter contains several binding sites to facilitate regulation upon TCR activation. TCR stimulation activates the NF-κB pathway, leading to the binding of the NF-κB complex to the *IRF4* gene promoter and activating its transcription [46]. In particular, c-Rel, a subunit of NF-κB, is crucial for this event, as *c-Rel*-deficient lymphocytes are unable to upregulate *Irf4* expression in response to mitogen-mediated TCR stimulation [46]. DNA motif analysis revealed that the *IRF4* gene promoter also contains a FOXP3-binding site [43] and gamma-activated site (GAS) sequences that can be bound and activated by STAT proteins such as STAT4 and STAT6 [7]. Interestingly, in DCs, the *IRF4* gene promoter also contains a binding site for the IRF4 protein itself, indicating an autoregulatory loop controlling its own expression [7].

Unlike in T cells, where IRF4 serves as a translator of TCR signal strength, in B cells, the B-cell receptor (BCR) serves as an upstream regulator of IRF4 transcription [8]. Additionally, signaling pathways, including CD40, IL-4 [5], and pre-BCR pathways [47], as well as cytokine stimulation via the NF-κB pathway, have been implicated in the induction of *IRF4* expression in B cells [46]. In addition to signaling pathways, IRF4 is a direct target of PAX5, a master regulator of B-cell commitment [47].

#### 3.3.2. Post-Transcriptional Regulation of IRF4

At the post-transcriptional level, IRF4 activity is modulated by several mechanisms. It can be inhibited by IBP (IRF4-binding protein, also known as Def-6), an activator of the Rho GTPase Rac, which physically sequesters IRF4, inhibiting its activity [48] in CD4^+^ T cells [49] and B cells [50]. IRF4 activity can also be repressed by its interaction with FK506-binding protein 52 (FKBP52), which interferes with the DNA-binding ability of IRF4 [51]. In contrast, IRF4 can be phosphorylated by ROCK2, which leads to transactivation of target genes such as *IL-17* and *IL-21* [49]. This phosphorylation event can be impeded by IBP-mediated IRF4 inhibition. These various findings highlight the upstream pathways that intricately orchestrate IRF4 expression in immune cells.

### 3.4. IRF4 as a Transcriptional Repressor

Notably, IRF4 has also been reported to act as a transcriptional repressor. The binding of IRF4 itself or IRF4 in a heterodimer with IRF8 to ISREs can repress the transcription of interferon-inducible genes and inhibit IRF1-mediated gene transcription in T cells and macrophages [10,52]. In B cells, IRF4 binds to the *BCL6* promoter and suppresses its expression, leading to the terminal differentiation of B cells [53]. The binding of IRF4 to the BCL6 protein also blocks the transactivating ability of IRF4 [5], thus suggesting negative feedback regulation between the *IRF4* and *BCL6* genes. Additionally, IKZF1 and IRF4 repress a subset of genes, including *Ebf1* and *Haao,* during plasma cell differentiation [45].

## 4. Oncogenic Mechanisms Mediated by *IRF4*

### 4.1. Activation of IRF4 in Mature Lymphoid Neoplasms

In addition to its roles in normal development and immunity, IRF4 has been implicated as an oncogene in mature lymphoid neoplasms. *IRF4* was originally identified as the *MUM1* gene from the chromosomal translocation t(6;14)(p25;q32) involving the *IgH* locus in MM cells [9]. Subsequent studies have demonstrated that *IRF4* is highly expressed in various types of both B-lymphoid and T-lymphoid neoplasms, including multiple myeloma (MM) [9,54,55,56,57,58,59,60], diffuse large B-cell lymphoma (DLBCL) [61,62,63,64,65,66,67,68,69,70], Burkitt’s lymphoma (BL) [71,72], follicular lymphoma (FL) [61,73,74], anaplastic large-cell leukemia (ALCL) [75,76,77,78,79,80,81], adult T-cell leukemia/lymphoma (ATL) [10,82,83,84,85,86,87,88,89], and mycosis fungoides (MF) [75,77]. Accordingly, various studies have demonstrated the functional requirement of IRF4 for cancer cell survival and proliferation. Forced expression of IRF4 can increase anchorage-independent colony-forming properties in rat fibroblasts [9]. Genetic knockdown of *IRF4* or treatment with IRF4-degrading immunomodulatory drugs (e.g., lenalidomide or pomalidomide) hinders cell proliferation and induces apoptosis in various lymphoid neoplasms in vitro [57,58,59,67,68,82,90,91,92,93]. Additionally, our recent study using zebrafish demonstrated that *IRF4* overexpression in lymphocytes induces the T-cell lymphoma phenotype, which can be accelerated in the *p53* mutant background [94]. 

**Figure 1:**
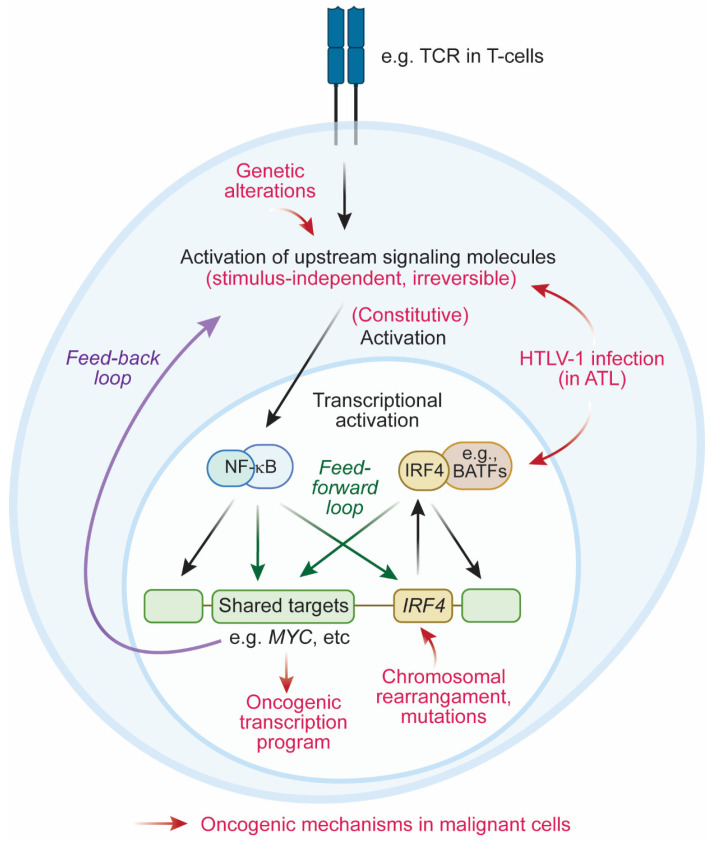
Signaling pathway and molecular mechanisms that activate IRF4 in malignant cells. In normal T cells, MHC–TCR interaction activates upstream intracellular signaling pathways, which leads to the activation of NF-κB. Similarly, in normal B cells, receptor signaling (e.g., BCR) leads to the activation of NF-κB. NF-κB in turn transcriptionally upregulates *IRF4*, which induces critical immune cell responses. This mechanism is thought to be stimulus-dependent and, thus, reversible. In contrast, in malignant lymphocytes (e.g., DLBCL, ALCL, and ATL), the NF-κB–IRF4 pathway is often constitutively activated in a stimulus-independent manner due to several oncogenic mechanisms (red arrows). Genetic alterations in the upstream signaling pathway can constitutively activate NF-κB. HTLV-1 infection, which is observed specifically in ATL cells, can also activate NF-κB and induce the expression of interacting partner of IRF4 (i.e., BATFs). IRF4 gene expression can also be aberrantly induced via chromosomal rearrangements (e.g., chromosomal translocation and amplification). IRF4 protein function may also be altered due to genetic mutations. These mechanisms lead to the constitutive activation of NF-κB and/or IRF4, which induces the oncogenic transcription program. Furthermore, NF-κB and IRF4 activate the upstream signaling pathways via a feedback loop and also regulate the same targets (e.g., *MYC*) via a coherent feedforward loop, which further enhances the transcription program.

Given the oncogenic advantage conferred by IRF4 overexpression, a plethora of genetic mechanisms that aberrantly activate IRF4 exist, ranging from chromosomal translocations to genetic amplifications, mutations, and constitutive activation of upstream signaling pathways (Figure 1). For instance, the *IRF4* gene was found to be translocated with the *IgH* regulatory element in MM and DLBCL cases and with the *TCRA* locus in ALCL cases [9,54,63,64,76], which induces aberrant expression of *IRF4* under a highly active transcriptional regulatory element. Another potential mechanism of *IRF4* overexpression includes constitutive activation of NF-κΒ, which is an upstream factor of IRF4. In DLBCL and ATL cases, genetic mutations in NF-κΒ pathway components (e.g., *A20/TNFAIP3* and *CARD11)* have been reported [65,66,87]. In the case of ATL, NF-κΒ signaling is also activated by the Tax onco-viral protein induced by HTLV-1 retroviral infection [89,95,96], while BATF3, a binding partner of IRF4, is induced by HBZ, which is also encoded by HTLV-1 [92]. Aside from aberrant overexpression, common genetic mutations in the DNA-binding domain of *IRF4* have been reported in MM and ATL cells [55,56,87] and are thought to provide IRF4 with stronger transcriptional activity in the nucleus [97].

### 4.2. Oncogenic Transcription Programs Driven by IRF4

#### 4.2.1. MYC Is a Common Downstream Target of IRF4

IRF4 exerts its oncogenic effects through the modulation of critical oncogenic transcriptional regulatory networks in mature lymphoid neoplasms. Although most networks are specific to the cellular context, a common recurring finding is a correlation between the expression of *IRF4* and the *MYC* oncogene, as observed in both B-cell and T-cell malignancies.

In MM, *MYC* has been established as a downstream target of *IRF4*. Chromatin immunoprecipitation-sequencing (ChIP-seq) analysis confirmed the binding of IRF4 to the *MYC* gene locus in MM cells, indicating that *MYC* is transcriptionally regulated by IRF4 in these cells [57]. MYC also regulates the *IRF4* gene in MM cells, thereby forming a positive feedback loop [57,98]. Activated B-cell (ABC)-DLBCL cells also express high levels of IRF4 and MYC [67]. Similar to the case in B-cell neoplasms, MYC is one of the key downstream target genes of IRF4 in several mature T-cell neoplasms, including ALCL [91] and ATL cells [82,92]. Our recent study using a transgenic zebrafish model also demonstrated that MYC is commonly upregulated in IRF4-induced T-cell lymphoma [94]. 

#### 4.2.2. IRF4 Is an Oncogenic Master Transcription Factor That Characterizes Tumor Type

Notably, IRF4 also regulates many genes that are specifically expressed in and characterize each tumor type. For example, IRF4 regulates CCL5 production in HL cell lines [99]. This effect can be reversed by CD40 engagement and the subsequent upregulation of *IRF4* expression [99]. Forced expression of *IRF4* in *IRF4*-negative Burkitt’s lymphoma cell lines (Daudi and Raji) upregulates plasma cell marker genes, such as *CD38* and *CD138,* and downregulates GCB cell marker genes, such as *BCL6* and *PAX5*. The expression of *Blimp-1*, which inhibits *MYC*, and *XBP1* is also increased, together with changes in *CD38, CD138*, *BCL6*, and *PAX5* expression [100], implying differentiation of B cells toward plasma cells. Similarly, in ATL cells, IRF4 regulates *CCR4* and *CD25/IL2RA*, which are known marker genes of Tregs and, thus, reflect the origin of ATL cells [82].

Of note, many recent studies have demonstrated that genes that characterize each cell type are often regulated under super-enhancers, a cluster of enhancers defined by active histone marks [101]. In many cancers, super-enhancers are found at genes that reflect cellular status, as well as functionally important genes such as oncogenes. In fact, our previous study with super-enhancer profiling for primary ATL cells showed that super-enhancers are associated with genes involved in T-cell function and the TCR activation pathway, including *CCR4* and *CD25/IL2RA*, as well as oncogenes, including *MYC* and *BIRC3* [102]. In addition, our recent study demonstrated that DNA-binding regions of IRF4 and NF-κB are enriched in super-enhancers; the majority of super-enhancers are bound by these two transcription factors [82]. IRF4 and NF-κB positively regulate many super-enhancer-associated genes and, thus, are required for the maintenance of ATL cells. Together, these results indicate that IRF4 can produce and sustain a unique transcriptional program that characterizes tumor type, thus implicating its role as an oncogenic master transcription factor.

#### 4.2.3. Feedback Loop between IRF4 and NF-κB

NF-κB is a critical upstream pathway of IRF4, which is often constitutively activated in various mature lymphoid neoplasms. Notably, IRF4 can in turn activate the NF-κB signaling pathway in several tumor types (Figure 1). In ABC-DLBCL, IRF4 forms a heterodimeric complex with SPIB that upregulates NF-κB signaling by inducing *CARD11* expression and blocking type I IFN signaling [67]. This IRF4–SPIB–CARD11 axis is essential for the survival of ABC-DLBCL cells, whereby a change in a single amino acid that mediates the interaction of IRF4 with SPIB inhibits cell growth [67]. IRF4 also forms a distinct positive regulatory loop with JMJD3 to activate NF-κB [103]. JMJD3 activates IRF4 expression, mediating the antiapoptotic effect of JMJD3 in ABC-DLBCL cells. IRF4 reciprocally stimulates JMJD3 expression to form a positive feedback loop, which further amplifies NF-κB activation. As expected, this IRF4–JMJD3–NF-κB loop increases IRF4 expression and promotes survival in these cells [103].

A similar type of positive feedback loop has also been demonstrated by us for ATL cells [82]. As mentioned above, IRF4 and NF-κB bind to the majority of super-enhancers, many of which are associated with genes involved in the TCR–NF-κB pathway [102]. IRF4 positively regulates many of these genes and, thus, can further activate the NF-κB pathway.

### 4.3. Coherent Feedforward Loop Mediated by IRF4 and NF-κB

In addition to the feedback activation loop between NF-κB and IRF4, our recent study also highlighted another regulatory structure by which IRF4 contributes to the maintenance of the gene expression program in ATL cells [82]. Our ChIP-seq analysis demonstrated that IRF4 and NF-κB co-occupy the same regulatory elements of downstream target genes at a high frequency. RNA-seq analysis revealed that the genes positively regulated by IRF4 are also positively regulated by p65, a subunit of NF-κB, while the genes positively regulated by p65 are also positively regulated by IRF4. Thus, IRF4 and NF-κB regulate downstream target genes in the same direction. The shared targets include oncogenes, such as *MYC,* as well as many genes in the TCR–NF-κB pathway.

Hence, in this instance, the top-tier transcription factor NF-κB regulates the second-tier transcription factor IRF4, and both of them share and coordinately regulate the same downstream genes, which in turn further activates the upstream TCR–NF-κB pathway. This structure is called a coherent-type feedforward loop (Figure 1). Although further investigation is necessary, previous studies using model systems suggested that this type of loop can serve as a “detector” to rapidly respond to the input signal and to avoid fluctuations in output expression, thereby increasing the robustness of the cellular state [104]. Thus, the feedforward loop mediated by IRF4 and NF-κB is likely required for the maintenance of the oncogenic transcriptional program in malignant lymphocytes.

## 5. Discussion and Future Directions

In this review article, we described the molecular functions of *IRF4* in mature lymphoid neoplasms. This gene has a unique history in biomedical research, which has provided many significant insights into immunology, molecular biology, and oncology. IRF4 exerts its oncogenic effects by forming specific regulatory loops and regulating downstream targets, many of which are essential for normal lymphocyte functions and characterize tumor type. All these findings identify *IRF4* as an oncogenic master transcription factor.

One important notion is that, under normal conditions, *IRF4* is physiologically expressed in mature lymphocytes in a stimulus-dependent manner. However, malignant cells constitutively express *IRF4* at a higher level than their normal counterparts. Thus, precancerous clones that have acquired a high level of *IRF4* expression in an irreversible manner (i.e., due to genetic mutations of the *IRF4* gene or members of its upstream signaling pathway) would have a clonal advantage to expand (Figure 1). For example, NF-κB is constitutively activated in ATL cells due to somatic mutations in members of the TCR–NF-κB pathway and persistent infection with HTLV-1 retrovirus, leading to sustained expression of *IRF4*. In addition, IRF4 and NF-κB form a multilayered coherent feedforward loop, which likely contributes to the amplification and/or maintenance of high-level expression of *IRF4*, as well as its downstream targets. This regulatory circuit is in marked contrast to IRF4 expression in normal T cells, in which IRF4 is transiently induced upon TCR activation.

Given that many cancer cells are highly dependent on IRF4, the inhibition of this protein would be an ideal therapeutic approach. Although IRF4 is an essential mediator of immunity, its expression in normal immune cells is transient; thus, the effect of IRF4 inhibition could be reversible. However, a specific inhibitor of IRF4 has not yet been developed. In general, transcription factor proteins are difficult to target with small-molecule inhibitors. Additionally, IRF4 requires other transcription factors for DNA binding, depending on the cell type. Thus, it would be challenging to design an inhibitor that blocks the DNA-binding ability of specific IRF4 heterodimers. Nevertheless, the activity and expression of IRF4 can still be modulated by regulating the mechanisms upstream of IRF4. As an example, the use of immunomodulatory derivative (IMiD) compounds that can reduce *IRF4* expression is a feasible approach and has already been tested in MM and other lymphoid neoplasms. Inhibition of the upstream NF-κB pathway could be another approach, although it has been shown to cause several major toxicities in animal models [105]. Another study showed the efficacy of antisense nucleotides targeting IRF4 in MM cells [106]. Additionally, our recent studies showed that IRF4 transcription is highly sensitive to inhibition of the general transcriptional machinery. Treatment with a small-molecule CDK7 inhibitor significantly reduced the expression of *IRF4* in ATL cells in vitro [82]. IRF4-driven zebrafish T-cell lymphomas also respond to a small-molecule BRD4 inhibitor in vivo [94]. Hence, these studies provide evidence supporting transcriptional inhibition of IRF4 as a potential therapeutic option.

## 6. Conclusions

In conclusion, numerous studies have demonstrated the importance of IRF4 in normal development, immunity, and cancers. IRF4 serves as a master transcription factor that regulates the essential transcription program that is unique to each cellular context. Targeting this factor is a feasible approach to disrupt critical oncogenic mechanisms in mature lymphoid neoplasms.
